# 

**Published:** 1903-04-18

**Authors:** 


### NOTICE TO CORRESPONDENTS.

All MSS., Letters, Books for Review, and other matters intended for the Editor should be addressed THE EDITOR, "THE HOSPITAL," THE HOSPITAL BUILDINGS, 28 & 29 SOUTHAMPTON STREET, STRAND, W C.

The Editor cannot undertake to return rejected MS., even when accompanied by stamped directed envelope.

### Notice to Advertisers and Subscribers.

All Advertisements, Orders for Copies of THE HOSPITAL, and Business Communications should be addressed to **THE MANAGER, (Not to the Editor)**, THE HOSPITAL, 28 & 29 Southampton Street, Strand, London, W.C.

The Cost of Subscription, post free, is as follows.—Per week, 3½d.; per quarter, 3s. 9d.; per half-year, 7s. 6d.; per annum, 15s. Foreign and Colonial:—Per annum, 19s. 6d., payable, in all cases, in advance.

**NOTICE.—Vol. XXXIII. of "The Hospital" (October 1902 to March 1903 in handsome cloth binding, will shortly be on sale at the office of this Journal, price 7s. 6d. Cases for binding the Numbers contained in this Volume 1s. 6d. Index to Vol. XXXIII., 2½d., post free.**

**The monthly part for April is now ready. Price 6d., by post 8d.**

### BOOKS RECEIVED.

Longmans, Green and Co.

"Colonial and Camp Sanitation." By G. V. Poore, M.D.

Gee and Co.

"Medical Practitioners' Accounts." By J. H. May.

The General Medical Council.

"The Dentist's Register, 1903."
"The Medical Register, 1903."

Baillière, Tindall and Cox.

"Refraction of the Eye." By Kenneth Campbell.
"Operative Surgery." By Herbert W. Allingham.

Cassell and Co.

"Diseases of the Skin." By Malcolm Morris.

P. Blakestons Sons and Co.

"Materia Medica for Nurses." By J. E. Groff.

New York Medical Record.

"Renal Decapsulation for Chronic Bright's Disease." By G. M. Edebohls.

Smith, Elder and Co.

"Diseases and Injuries of the Eye." Sixth Edition. By Arnold Lawson.

Humanitarian League.

"Food and Fashion: Some thoughts on what we eat and what we hear."

W. Blackwood and Sons.

"Open-air Treatment." By Isabella Mears.

### THE STUDENT OF MEDICINE.

### APPOINTMENTS.

**George Ashton**, M.B., Ch.B.Vict., M.R.C.S.Eng., L.R.C.P. Lond., appointed Assistant Surgical Officer to the Manchester Royal Infirmary. **T. G. Beckett**, L.R.C.P.&S.Edin., L.S.A., appointed Honorary Medical Electrician to the Alfred Hospital, Melbourne. **E. H. Blake**, L.R.C.P.I., L.S.A.Lond., appointed Clinical Assistant to the Chelsea Hospital for Women. **C. V. Bourke**, M.B., Ch.M.Syd., appointed Honorary Assistant Gynæcologist to the Sydney Hospital, New South Wales. **John Charlton Briscoe**, M.B.Lond., M.R.C.P.Lond., appointed Assistant Physician to the Evelina Hospital for Children. **William Broad**, M.B., B.Sc.Glas., appointed Visiting Medical Attendant to the Warangesda Aborigines Station, New South Wales. **R. W. S. Christmas**, M.R.C.S., L.R.C.P.Lond., appointed District Medical Officer of the St. Neot's Union. **Philip S. Clarke**, M.B., appointed Resident Medical Officer to the Children's Hospital, Adelaide. **G. A. Fischer**, M.B., B.S., appointed Honorary Surgeon to the Department of Diseases of the Ear and Throat of the Adelaide Hospital. **E. M. Glanville**, M.B., appointed Medical Officer of the Casualty Department of the East London Hospital for Children, Shadwell. **Eleanor R. Henderson**, M.B., Ch.B Edin., appointed Surgeon to the Outdoor Department of Leith Hospital. **W. R. Jordan**, M.D.Lond., appointed Visiting Physician to the Birmingham Workhouse Infirmary. **A. S. Joske**, M.B. Ch.B Melb., appointed Honorary Surgeon to the Children's Outdoor Department of the Alfred Hospital, Melbourne. **R. A. Macleod**, M.A., M.B., C.M.Edin., appointed Assistant Resident Medical Officer in Mill Road Infirmary, Liverpool. **B. H. Morris**, M.B. B.S., appointed Medical Officer to the Yatala Labour Prison, South Australia. **W. Parsons**, M.R.C.S.Eng., L.R.C.P.Lond., appointed Assistant Medical Officer to the Islington Parish Infirmary. **J. M. Petrie**, M.B.Aber., D.P.H.Lond., appointed Clinical Assistant to the Chelsea Hospital for Women. **C. R. Player**, M.B., Ch.B.Melb., appointed Honorary Physician to the Children's Outdoor Department of the Alfred Hospital, Melbourne. **W. H. Read**, M.B., Ch.M.Syd., appointed Registrar and Anæsthetist to the Hospital for Sick Children, Sydney, New South Wales. **J. D. Richmond**, M.B., B.S.Glas., appointed Medical Officer of the Walton Workhouse of the West Derby Union. **E. R. Roseby**, M.B., Ch.M.Syd., appointed Government Medical Officer and Vaccinator at Nyngan, New South Wales. **G. A. W. Spear**, M.R.C.S., L.R.C.P.Lond., appointed District Medical Officer of the Petworth Union. **Esther M. Stuart**, M.B., C.M.Edin., D.P.H., B.Hy.Durh., appointed Female Medical Officer to the Newcastle-on-Tyne School Board. **Charters J. Symonds**, M.S., F.R.C.S., appointed Examiner in Surgery to the London University. **H. A. L. Willis**, L.R.C.P.Lond., M.R.C.S.Eng., appointed Government Medical Officer and Vaccinator at Gunning, New South Wales. **J. R. Woods**, appointed House Surgeon to the East London Hospital for Children, Shadwell.

### "The Hospital" Institutional Library.

"Hospitals and Asylums of the World." 4 Vols., with a Portfolio of Plans, complete £12 12s.

"Burdett's Hospitals and Charities, 1902." 5s.

"Burdett's Official Nursing Directory, 1903." 3s. net.

"Cottage Hospitals, General, Fever, and Convalescent." 10s. 6d.

"Uniform System of Accounts for Hospitals and Public Institutions." New Edition. 4s. net.

"Hospital Expenditure: The Commissariat." 2s. 6d.

"A Legal Handbook for the Use of Hospital Authorities." 2s. 6d.

"The Cottage Hospital Case Book or Register of Patients." 10s. and 12s. 6d.

"The Furnishing and Appliances of a Cottage." 6d.

All these are published by the Scientific Press, Ltd., and may be obtained through any bookseller, or direct from the publishers, 28 and 29 Southampton Street, Strand, London, W.C.

### SUCCESS.

Efficiency is the keynote of success in any profession, but especially is it so in the Nursing Profession. When one recollects the enormous strides that have been made in this important calling within the last fifty years, the truth of this statement is fully borne out. To attain efficiency, and its accompanying success, it is essential that the nurse should keep abreast with all that is new and up-to-date in the many branches of her profession. As a means to this end, the works of recognised and competent teachers will be found of the greatest assistance.

It is the object of The Scientific Press to supply nurses with the best and latest manuals on Nursing and Allied Subjects, and we append a selected list of some of the most valuable books.

### NURSING.

**THE NURSING PROFESSION: HOW AND WHERE TO TRAIN.**
By Sir Henry Burdett, K.C.B.
Price 2/- net; post free, 2/4.

**A HANDBOOK FOR NURSES.**
By J. K. Watson, M.D., M.B., C.M.
New Edition. Price 5/- net; post free, 5/4.

**NURSING: ITS THEORY AND PRACTICE.**
By Percy G. Lewis, M.D., M.R.C.S., L.S.A.
New Edition. Price 3/6 post free.

### SURGICAL WARD-WORK.

**SURGICAL WARD-WORK AND NURSING.**
By Alex. Miles, M.D.Edin.
Second Edition. Price 3/6 net; post free, 3/10.

### ANATOMY.

**ELEMENTARY ANATOMY AND SURGERY FOR NURSES.**
By Wm. McAdam Eccles, M.S.
Price 2/6 post free.

### PHYSIOLOGY.

**ELEMENTARY PHYSIOLOGY FOR NURSES.**
By C. F. Marshall, M.D., B.Sc., F.R.C.S.
Price 2/- post free.

### MASSAGE.

**ART OF MASSAGE.**
By Mrs. A. Creighton Hale.
Second Edition. 6/- post free.

### MIDWIFERY.

**A PRACTICAL HANDBOOK OF MIDWIFERY.**
By Francis W. Haultain, M.D.
New and Revised Edition. 6/- net; post free, 6/3.

**THE NURSE'S DICTIONARY OF MEDICAL TERMS AND NURSING TREATMENT.**

Compiled for the use of Nurses. By Honnor Morten.

Fourth and Revised Edition. Demy 16mo. (suitable for the apron pocket), bound in cloth boards, 160 pp., 2/- post free; in handsome leather, gilt, 2/6 net; 2/8 post free.

*A Complete Catalogue will be forwarded Post Free on application.*

THE SCIENTIFIC PRESS, LIMITED, 28 & 29 Southampton Street, Strand, London, W.C.

